# Green and sustainable fabrication of DES-pretreated high-strength densified wood

**DOI:** 10.1007/s00226-024-01594-7

**Published:** 2024-08-27

**Authors:** Akash Madhav Gondaliya, Mahfuzul Hoque, Sreenath Raghunath, E. Johan Foster

**Affiliations:** Chemical and Biological Engineering, University of British Columbia, Vancouver, Canada

## Abstract

Wood is a sustainable, benign, and high-performing green structural material readily available in nature that can be used to replace structural materials. However, insufficient mechanical performance (compared to metals and plastic), moisture sensitivity, and susceptibility to microorganism attack make it challenging to use wood as it is for advanced engineering applications. We here present an efficient approach to fabricating densified wood with minimal time and waste generation, demonstrating high mechanical strength, and decreased water penetration on the surface. Wood slabs were treated with deep eutectic solvents (DESs) to solubilize the lignin, followed by in-situ regeneration of dissolved lignin in the wood. Then, the slabs were densified with heat and pressure, turning the wood into a functionalized densified material. Lignin regeneration and morphological changes were observed via two-photon microscopy and Scanning Electron Microscopy (SEM), respectively. The final product is less susceptible to water absorption on the surface and has enhanced flexural strength (> 50% higher), surface hardness (100% increased), and minimal set recovery compared to natural wood. The improved mechanical performance is due to regenerated lignin which acts as a glue and fills spaces present within the interconnected cellulose network inside the wood, forming a highly dense composite during densification. Such enhancement in the properties of DES-densified wood composite makes it a favorable candidate for advanced structural and engineering applications.

**Supplementary Information:**

The online version contains supplementary material available at 10.1007/s00226-024-01594-7.

## Introduction

High-strength structural materials, such as concrete (D’Amico et al. 2021), metal/alloys (Huo et al. 2014), carbon fiber (Jiang et al. 2017; Singh et al. 2021), polymers (Li et al. 2019), etc., have gained huge importance in industrial and construction applications. All these structural materials harm the environment due to complex and harsh manufacturing processes (Shi et al. 2015; Shirvanimoghaddam et al. 2017; Topping et al. 2012). For example, to manufacture metals, special conditions such as super high temperatures (up to 1800º C), inert atmospheric conditions, or even a vacuum environment are required, which not only consumes a lot of energy while processing but also pollutes the environment (UN Environment 2017). To tackle the issue, it is crucial to manufacture green, sustainable, easy-to-process, and low-cost structural advanced materials that will lead to reduced energy consumption. For the same, the World Green Building Council in 2019 announced achieving a carbon neutrality target by 2050 (Embodied Carbon 2019).

Due to its low cost, lightweight features, and excellent mechanical properties, wood has been used for thousands of years to make structural materials in construction and furniture. However, for advanced structural applications, especially outdoor applications, natural wood is not ideal compared to composite materials, concrete, and metals (Cabral et al. 2022) due to its susceptibility to moisture, limited dimensional stability, low hardness and wear resistance, poor resistance to bio-deterioration from fungi, termites, and marine borers, as well as its low resistance to ultraviolet (UV) radiation (Sandberg et al. 2021). Therefore, research has been done on wood modifications to improve the overall properties of wood (Chen et al. 2021; Han et al. 2019; Li et al. 2016; Song et al. 2018; Wei et al. 2019; Xiao et al. 2021). Since the mechanical performance of wood is directly dependent on the density, previous studies have employed chemical treatment (delignification) along with physical modifications (densification) to manufacture wood composites as strong as some industrially used alloys (Ellouze et al. 2020; Frey et al. 2018; Guan et al. 2018; Jia et al. 2017; Jirouš-Rajković and Miklecić 2019; Li et al. 2018; Song et al. 2018a; Xiao et al. 2021).

In most of the research on wood modification, lignin removal was crucial pretreatment, and it was done using an aqueous solution of sodium chlorite (Montanari et al. 2019) or an aqueous solution of sodium hydroxide and sodium sulfite (Shi et al. 2015). These studies have shown that partial delignification from natural wood, specifically removing lignin between cell walls, increases the pores and makes the wood less rigid, which leads to improvement in wood densification (Gondaliya et al. 2023a, b; Song et al. 2018). However, in preparing all these biobased products, a large quantity of residual waste liquid/black liquor is generated in the pretreatment process, especially delignification (consisting of chemicals such as sodium sulfite, enzymatic degradation, sodium hydroxide, organic solvents like methanol, and, alkaline hydrogen peroxide) (Ali et al. 2020; Guan et al. 2018; Song et al. 2017; Van Den Bosch et al. 2015) which is hard to recycle. Moreover, toxic gases are also released in these processes; for instance, chlorine gas is released during the delignification using sodium chlorite, which is detrimental to human health (Fan et al. 2022). Some organic solvents are volatile, toxic, and hurt the environment. Special chemicals like hydrogen peroxide need to be carefully stored and handled in sealed, lightproof, cold, and strict environments due to their strong oxidizing nature (Fan et al. 2022). These factors of delignification chemicals, such as cost, scalability issues, and efficacy, obstruct their application in large-scale production, which in turn weakens the sustainability factor of wood-based products. Furthermore, the chemical hydrolysis to remove lignin also affects cellulose’s crystallinity, leading to a decrease in mechanical properties (Xiong et al. 2019).

Hence, there is a need to find environmentally sustainable, and economically viable solutions to pretreat wood to produce advanced engineering products such as roof tiles, cladding decking, and outdoor flooring. Recently, a new method of solubilizing lignin has been proposed using a sub-class of ionic liquids, that is, deep eutectic solvents (DESs) (Francisco et al. 2012). DESs are in the liquid state solution at room temperature, formed by the combination of hydrogen-bond acceptors and hydrogen-bond donors via strong hydrogen-bond interaction. They exhibit various advantages over other chemicals, such as being environmentally friendly, (X. Li and Row 2016; Dai et al. 2013) economical (Dai et al. 2013) (versus traditional ionic liquids), easily available precursors, and biodegradable (Bi et al. 2018). We have used the choline chloride and lactic acid combination to prepare DES in our study, which has provided evidence to solubilize lignin and hemicellulose from wood selectively (Alvarez-Vasco et al. 2016; Fan et al. 2022; Li et al. 2016; Ran et al. 2023; Wu et al. 2020a, b). The reaction conditions and overall process are mild and do not hamper the crystallinity of cellulose fibers (Bi et al. 2018). Previously, DESs have been used to delignify the wood before densification; although the recovery of DESs is possible however, it possesses certain challenges in separation, such as the energy-intensive distillation process to separate water from DES solution.

In the densification process, the lumens in the wood are compressed radially to reduce the volume fraction of empty lumens, making the wood uniform. Densification also leads to formation of hydrogen bonds with the adjacent cell wall, hence increasing the load-bearing properties (Luan et al. 2022). However, previous literature studies have reported that certain challenges are associated with densified wood post-densification treatment, such as the springback effect in direct contact with water or the presence of a high-humidity environment and excessive densification time (~ 24 h) (Fan et al. 2022; Sandberg et al. 2021). To overcome these challenges post-treatment, such as high heat, steam, coating with polyurethane (PU) (Song et al. 2018b), and impregnating with thermosetting resins (Lykidis et al. 2019; Schwarzkopf 2021) in addition to densification can be done to increase the hydrophobicity and reduce the set recovery of densified wood. However, high-temperature treatment can lead to a loss in mechanical performance (Gong et al. 2010; Kutnar and Kamke 2012), and formaldehyde chemical in the thermoset resin (MF-melamine formaldehyde and PF-phenol formaldehyde) poses significant environmental concerns (Wu et al. 2020a, b). Our previous work addressed these issues of excessive densification time, post-treatment and springback effect by impregnating a biobased binder (Polylactic Acid) in the wood and carrying out in-situ polymerization and densification (Gondaliya et al. 2023a, b). However, we still need to carry out delignification to create extra pockets in the wood for the polymer impregnation, which leads to the generation of lignin waste and required additional resources.

Therefore, to address the issue of lignin waste generation, reducing densification time, and the springback effect; herein, we have developed the process of completely removing the delignification step and have shown that lignin removal is not necessary to get complete densification and achieve a densified product. We employed a sustainable DES solution to solubilize lignin in the wood and regenerate that dissolved lignin by using an antisolvent, i.e., water to distribute it throughout the wood fibers evenly. We hypothesize that this regenerated lignin aided in the densification process under pressure and temperature and acted as a glue, thereby increasing the bonding strength. To verify this hypothesis, we assessed and compared the dimensional stability and mechanical properties (using Instron to calculate Modulus of Rupture (MOR), Shore D hardness) of natural wood with DES-treated densified wood, and their microstructure (using electron microscopy), crystalline structure (using X-ray diffraction), chemical structure (using infra-red spectroscopy), and content of lignin distribution (using optical microscopy). The results of this research can highlight the new feasible process for wood densification. The approach of in-situ lignin regeneration eliminates the need to delignify and remove lignin, reducing the amount of black liquor produced. As natural glue was used in the process, the densification time was reduced, rendering savings in energy cost and processing time. Because of the hydrophobic and stiff nature of lignin, we were also able to get the enhancement in water-repellent (using contact angle tests) properties and surface hardness.

## Materials and methodology

### Materials

Commercial cedar wood was purchased from Home Depot, Canada. The wood was cut into blocks with the following dimensions: 100 mm (longitudinal) × 38 mm (tangential) × 7.5 mm (radial). The original wood samples were free of any cracks. Chemicals DL- lactic acid (90% (T), C_3_H_6_O_3_), and choline chloride (98%, C_5_H_14_CINO) were purchased from Sigma-Aldrich (Canada), and all chemicals were used as provided.

### DES solution preparation

DES solution was made by mixing choline chloride in DL-lactic acid in a 1:10 molar ratio. The solution was then stirred at 250 rpm for 30 min, followed by heating (Thermo Scientific hotplate) at 60 °C and mixing for another 30 min until it was homogeneous and crystal clear.

### DES lignin mobilization and regeneration process parameter optimization

The beaker (1000 mL) was filled with the prepared DES solution at room temperature, and the wood blocks were completely immersed in it. The beaker was then kept in the vacuum desiccator for vacuum impregnation. The desiccator was depressurized to remove all the air bubbles from the wood for 30 min with a vacuum pressure of − 0.09 MPa. Followed by releasing the vacuum and letting the system rest in atmospheric pressure for 30 min. Repeating the cycles for a total of 3 times to ensure the DES solution impregnates deep inside the wood. DES-impregnated wood was then covered in an aluminum foil and kept in the oven at 3 different temperatures (80, 100, and 120 °C) and treated for 3 different time variations (4, 8, and 12 h) for the preliminary analysis to study the mechanical performance. So, in total, there were 9 different treatments applied to the natural wood (NW) sample. The DES-treated samples were impregnated with reverse osmosis (RO) water as an anti-solvent and then washed to remove any excess DES from the wood surface and inside the lumens. The dimensional stability of wood treated at 100 °C for 8 h was exceptional compared to samples treated at 80 °C and 120 °C as evident from the MOR results; therefore, this treatment method was used in our further analysis, such as morphological, hardness, and optical microscopy.

### DES-treated densified wood

**Fig. 1:**
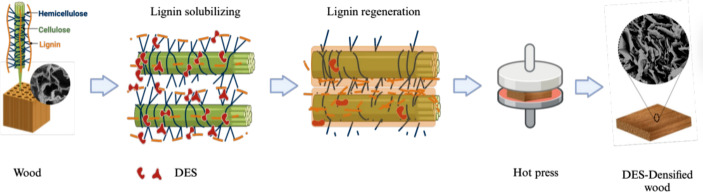
Schematic of synthesis of Deep Eutectic Solvent (DES) treated densified wood composite. DES-impregnated wood was kept in an oven to solubilize and depolymerize the lignin inside the wood at 100 °C and for 8 h. Finally, after a quick wash to remove any excess chemicals, the treated wood samples underwent the pressure and temperature-assisted densification process to manufacture the composite. “Image Created with BioRender.com”

The DES-treated samples were washed and impregnated with RO water to remove the residual DES solution and regenerate the lignin on the cellulose surface. Later, the washed samples were pressed using a hot press (Carver, USA) for 8 h at 120 °C using a 4 mm spacer between the hotplate, ensuring a similar degree of compression for each sample. Figure 1 shows the schematic of the process. For the compression springback test, the natural wood without DES treatment was also densified under the same conditions mentioned previously (8 h at 120 °C using a 4 mm spacer) as DES-treated densified samples.

### Scanning electron microscopy (SEM)

Scanning electron microscopy (model: Hitachi SU3500, Japan) was used for morphological analysis to observe the effect of DES treatment and to confirm the deposition of lignin on the surface. The solid wood sample had a dimension of 5 mm (L) × 5 mm (W) × 2 mm (H). The samples were carefully placed on the aluminum stub using carbon tape (conductive adhesive). Then the samples were sputter coated (8–9 nm) with Iridium under vacuum conditions using a sputter coater (model: Leica EM MED020, Germany).

### Laser scanning microscopy (LSM)

DES-induced surface (chemical) topography of wood samples was analyzed using a laser scanning microscope (model: Zeiss LSM 510 MP, Germany). A femtosecond (fs) laser (model: Coherent Chameleon, Germany) with tunable excitation wavelength (EX WL) from 680 to 980 nm and a laser pulse duration of 120 fs was employed as the light source. Two-photon excitation microscopy (2PEM) mode was chosen to investigate the wood samples over conventional laser scanning confocal microscopy (LSCM) for the following reasons:

- Non-descanned (NDS) light path (Crowe and Ellis-Davies 2014; Denk et al. 1990) afforded maximum collection efficiency of the scattered photons from the surface of the specimen — highest signal-to-noise (S/N) ratio.
- Deeper penetration (probing depth: 300–600 mm) to the thick wood samples.

During 2PEM investigations (NDS-detection), natural wood was excited with three different wavelengths, which are 710, 800, and 900 nm, to observe the selectivity of lignin in wood (see, Figure S4). Also, laser power was varied from 0.5 to 2% during the image acquisition. Three excitation wavelengths (710, 800, and 900 nm) were used to discover the suitable EX WL for the pristine wood samples. The exposure time was always kept short (i.e., 30 s) to minimize photodamage.

After the initial exploration, 710 nm was chiefly employed for the 2PEM-based imaging of the wood specimen as a function of laser power to underpin the role of the DES treatment. Besides NDS-detection, emission (fluorescence) signal was also collected using multi-channel detection mode — three different channels (ch) with bandpass (BP) of 435–485 (ch1), 480–520 (ch2), and 500–550 (ch3) nm, respectively.

### Chemical structure analysis: attenuated total reflectance-Fourier transform infrared spectroscopy (ATR-FTIR)

Fourier transform infrared (FTIR) spectroscopy (Bruker Inventio ATR-FTIR spectrometer, Germany) was employed to analyze the wood functional groups before and after treatment. The wood sample was finely ground into powder before scanning. Attenuated total reflectance (ATR) mode was used for recording the spectra. The scanning range was 400–4000 cm^− 1^, the scanning frequency was 10 kHz, and the scanning number was 3s (Figure S1).

### Mechanical performance test

A universal testing machine from Instron was used to find the maximum stress the sample could bear before it yielded. A 3-point bending setup was used following the ASTM D4761-19 with modification to obtain the stress vs. strain curve to determine the modulus of rupture (MOR) using the following equation.

$${\rm{MOR}}\,{\rm{ = }}\,{{3{\rm{*P*L}}} \over {2{\rm{*b*}}{{\rm{d}}^2}}}$$

where P is the maximum load applied, L is the span length, b is the width of the specimen, and d is the thickness of the specimen. The sample dimensions were 100 mm × 40 mm × 4 mm (L ×W × H) for the densified wood and 100 mm × 38 mm × 7.5 mm for the natural wood (Table S2). The samples were conditioned at 23 °C and 50% RH till constant weight was achieved before carrying out the test. The density of natural wood was 0.45 g/cc, and the DES-100-8 was 0.74 g/cc (Table S2). The span was pressed down along the center point of the sample (90 mm span length) perpendicular to the grain direction, with a loading speed of 10 mm/min. The difference in height between the natural wood (7.5 mm) and densified wood (4 mm) specimens resulted from the densification process, which inherently caused dimensional changes. Despite this, we ensured that all specimens were tested under the same span (90 mm) to maintain consistent testing conditions. While we acknowledge that the changes in thickness and width may influence the mechanical properties, our main objective was to compare the relative improvements in mechanical properties of DES-densified wood due to the DES-assisted densification treatment over the original natural wood sample.

### Surface hardness test

To quantify the surface hardness, a Shore D hardness tester instrument (model: Gain Express Holdings Ltd., Hong Kong) was employed. At least 5 different points from at least 4 samples for one treatment were randomly selected on the surface of natural as well as DES-densified wood. An average of all values was reported.

### Contact angle test

To study the water-repellency properties of the natural wood and DES-densified wood, a static contact angle test was conducted using the tensiometer (model: Theta Flex 300-Pulsating Drop 200, Biolin Scientific, Finland). The droplet size was kept at 5 µL. After releasing the drop, the contact angle was observed for at least 60 s. The contact angle (CA) measurements for the samples were taken over a 60-second period. Multiple tests (at least three) were conducted for each sample. From each test, the mean CA was calculated by averaging the left and right side CAs at time = 60 s. The final mean CA value was obtained by averaging these mean values from the three replicates.

### Lignin content determination

To determine the loss of lignin after the DES treatment we treated the natural wood with DES as reported in this paper (Section: DES Lignin Mobilization and Regeneration Process Parameter Optimization – DES-oven sample) for 4 h at 100 °C and for the comparison we also prepared a sample by boiling the wood piece in the DES solution (DES-boil sample). Wood boiling in DES has been reported in the previous literature to delignify the wood (Chen et al. 2019; Soares et al. 2021; Wu et al. 2020a, b). In our approach, we wanted to keep the maximum amount of lignin inside the wood. For the analysis, dry wood powder (sample: natural wood, DES-boil, and DES-oven) – *W*_i_ (Approx. 0.3 g) was used for the lignin content determination. The wood powder was soaked in ethanol at 20 °C for at least 3 h to remove extracts (e.g., fat, wax) from the wood. Then, sulfuric acid of 75% concentration (5 mL) was mixed with wood powder, and the mixture was left to sit for 2 h at room temperature. Later, distilled water was added to make the mixture, and the concentration of sulfuric acid was diluted to 3.0 wt% and the mixture was sealed and kept in an oven at 120 °C for 4 h. Finally, a Buchner funnel was used for performing vacuum filtration to separate the insoluble content and the content was washed using DI water. The insoluble material obtained after filtration was weighed after drying and was denoted by *W*_f_ (Weight final). The % lignin content was estimated using the following formula: [*W*_f_/*W*_i_] x 100%.

### Compression springback test

Compression Springback (CS) was evaluated to understand the shape-memory effects happening when the load (compression) is released after densification on DES-densified wood. The dimensions of the samples were measured before and after the densification process. CS was calculated using the following equation:

$$\:CS\:\left(\%\right)=\:\left[\frac{\left(tic-{t}_{t}\right)}{{t}_{i}-{t}_{t}}\right]*100\:$$

where ‘*t*_ic_’ is the thickness of the sample after densification, ‘*t*_t_’ is the predetermined compression thickness (4 mm-thickness of metal between hot plate), and ‘*t*_i_’ is the thickness of initial uncompressed natural wood.

### Crystallinity index (CrI %) determination

Powder X-ray diffraction (powder XRD) was performed on wood powder using a conventional benchtop diffractometer (model: D8-advance, Bruker, Germany). The samples were packed in a standard Bruker sample holder in a classic Bragg-Brentano geometry. To ensure, the highest signal-noise (S/N) for the specimen, a step size of 0.03° was set during the acquisition of the X-ray diffractograms (diffracted X-ray intensity) from 5° to 90° diffraction angle (Supplementary information, Figure S5). The crystallinity index (CrI %) was calculated using the Segal peak height method (Segal et al. 1959) by taking the ratio of crystalline (200) peak intensity at 2θ = 22.7° *(I*_*200*_*)* and intensity amorphous peak intensity *(I*_*am*_*)* at 18.6° diffraction angle (Supplementary information, Table S1). An equation to calculate CrI %:

$$\:CrI\:\left(\%\right)=\:\frac{{I}_{200}-{I}_{am}}{{I}_{200}}\:\times\:\:100$$

## Results and discussion

### Morphological characterization: scanning electron microscopy (SEM)

**Fig. 2:**
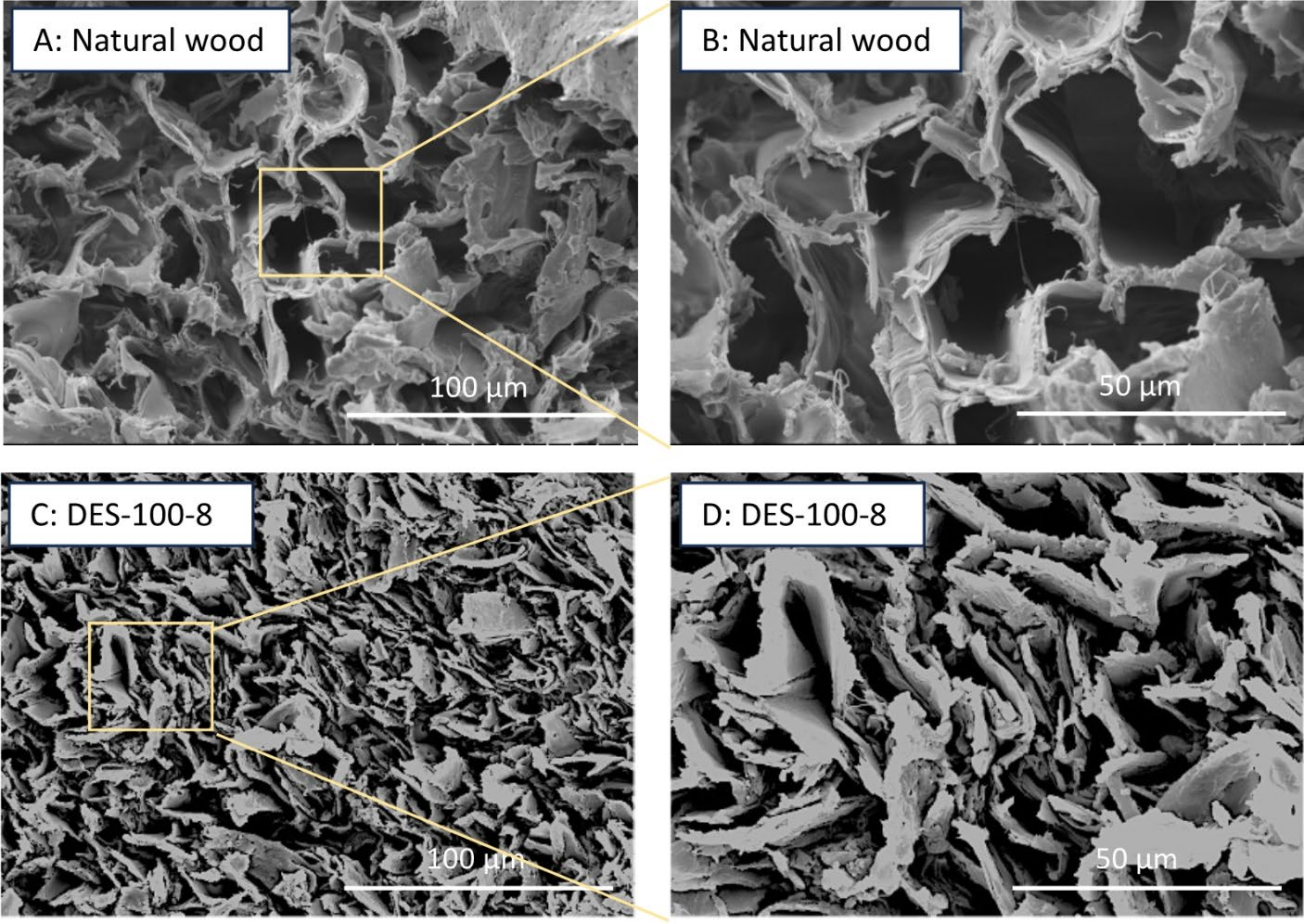
Cross-sectional SEM micrographs at low (500x) and high (1000x) magnifications for natural wood and DES-treated (labeled as DES-100-8) densified wood. 500x (**A**), and 1000x (**B**) for natural wood, and 500x (**C**), and 1000x (**D**) for DES-treated densified wood

Morphological analysis using SEM was carried out to observe the extent of densification. Figure 2 shows the cross-sectional SEM micrographs for the natural and DES-100-8 (DES-treated densified wood). Figure 2 illustrates open honeycomb structured channels, also referred to as ‘lumina” (Fig. 2A and B) in natural wood, while densification treatment led to the collapse of the open cell wall structure, removing any spaces between cell walls (Fig. 2C and D). Thus, in DES-100-8, complete bulk densification was achieved. Interestingly, these findings indicate that lignin removal (delignification) was not compulsory to achieve complete densification, which has been a crucial step in most of the previous densification studies (Fan et al. 2022; Gondaliya et al. 2023a, b; Song et al. 2018).

**Fig. 3:**
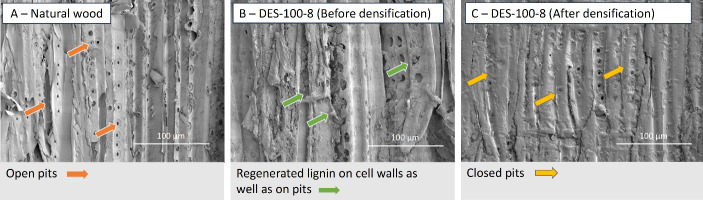
Surface SEM micrographs showcasing natural wood (**A**), DES-treated wood (DES-100-8) before densification (**B**), and DES-treated densified wood (DES-100-8) after densification (**C**)

**Fig. 4:**
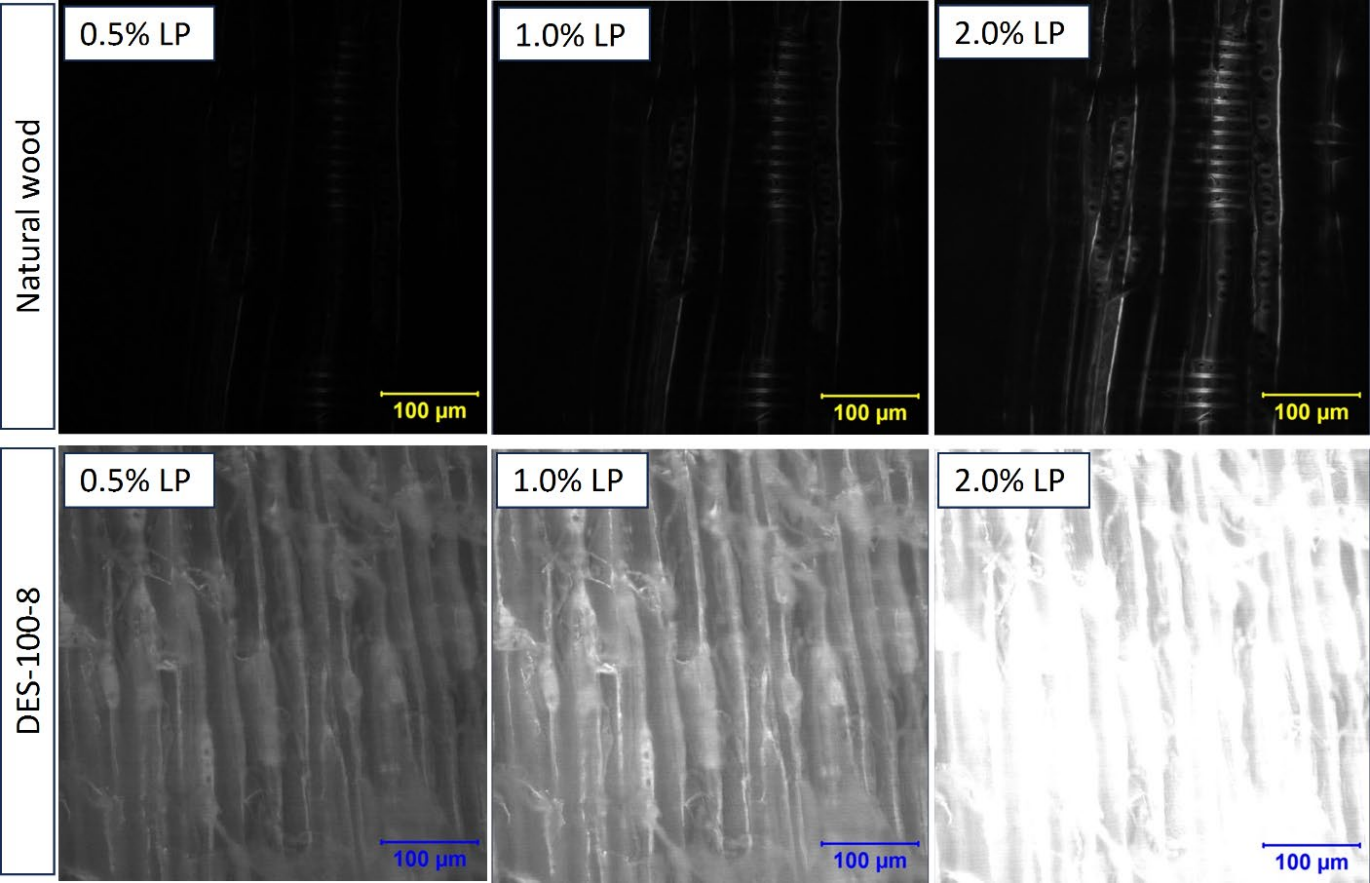
Optical (2PEM) micrographs of natural wood (denoted as NW) and DES-treated wood (denoted as DES-100-8) as a function of laser power (in %). (top) EX WL: 710 nm. Detection mode: Non-descanned (NDS). From left to right, the micrographs were captured with a gradual increment in laser power

**Fig. 5:**
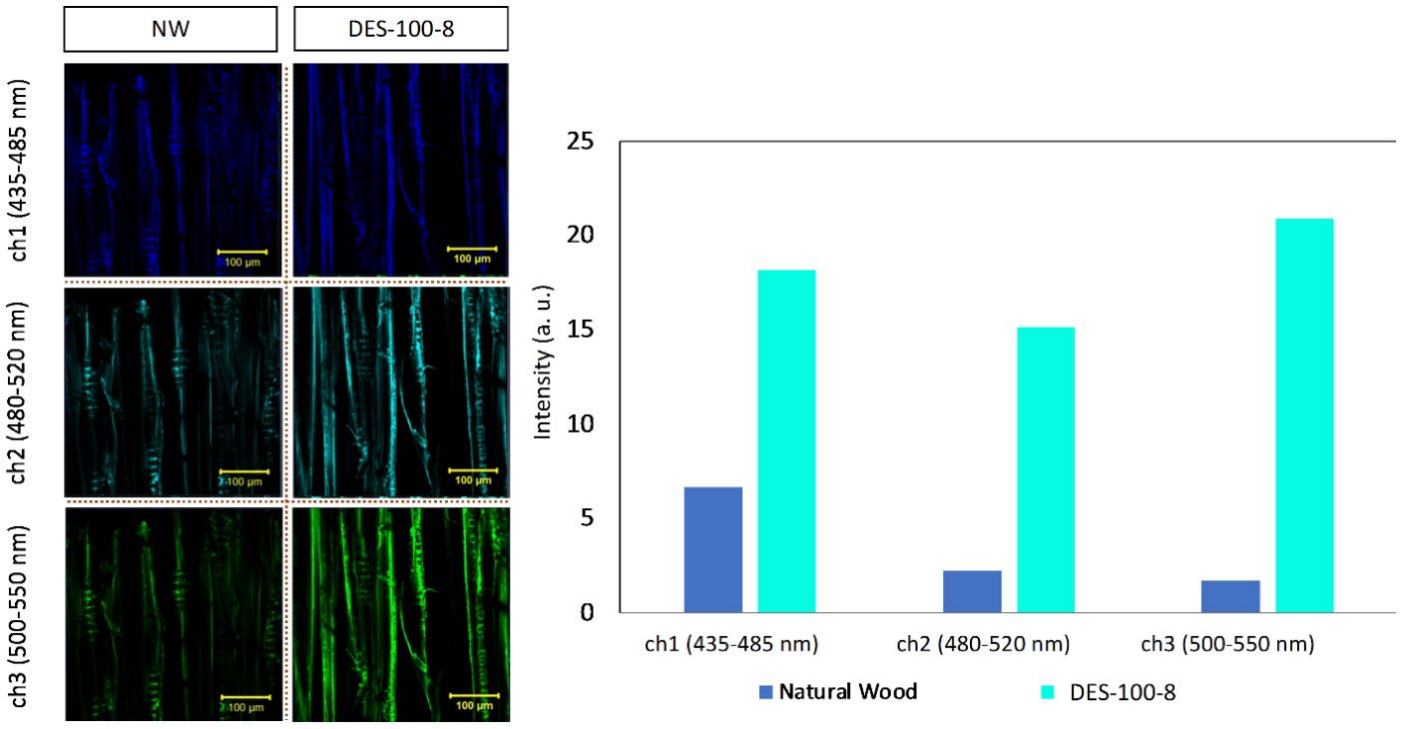
Optical (2PEM) micrographs of natural wood (NW) and DES-treated wood (DES-100-8) at 2.0% of laser power. (Left side) false color-coded micrographs (top-to-bottom) corresponding to ch1: 435–485 nm, ch2: 480–520 nm, and ch3: 500–550 nm, respectively. The (average) emission intensity is shown in the bar chart (right side). EX WL: 710 nm. Detection mode: multichannel

Next, Fig. 3 displays the surface SEM micrographs of the natural wood (A), DES-treated wood before densification (DES-100-8) (B), and DES-treated densified wood (DES-100-8 after densification) (C) to understand the effect of DES treatment and densification on the surface. Figure 3 (Image B) displays the regeneration of lignin particles on the cell walls covering the surface and, to some extent, clogging the pits. It is evident from Fig. 3 that the natural wood (A) has a lot of open pores (pits) on the surface marked with orange arrowheads. On the contrary, the DES-treated wood (B) surface shows a coat/layer of regenerated lignin, which is marked with green arrowheads, and (image C) DES-densified sample shows a smooth top surface with closed pores shown using yellow arrowheads. This is an interesting observation as we hypothesized that DES would solubilize lignin (partially) and then regenerate it when washed with antisolvent-like water (in this study). Similar regeneration of lignin particles has been reported in previous work as well (Guo et al. 2022; Xia et al. 2021). However, to confirm the lignin regeneration on the surface, optical microscopy-focused characterization was performed (*as shown in*, Figs. 4 and 5).

### Surface chemistry characterization: optical microscopy focused

To observe the lignin regeneration on the surface, laser scanning microscopy (LSM) was performed consisting of two analytical approaches, which are laser scanning confocal microscopy (LSCM) and Two-photon excitation microscopy (2PEM). Two-photon excitation microscopy (2PEM) requires simultaneous absorption of two photons by a fluorophore, which allows deeper penetration to the sample. Also, excitation is more efficient than confocal microscopy, which renders no benefits of pinhole aperture — *vis a vis*, the pinhole is completely opened in 2PEM during image acquisition. Thus, it allows for the capture of more light, which could be advantageous for visualizing the topographical alteration of fluorophore, i.e., lignin in chemically treated wood. We note that both LSCM and 2PEM have been prevalent in wood materials science and visualization of microstructure and topochemical variation (factors: pH, temperature, humidity, etc.) of softwood and hardwood (Donaldson et al. 2015; Thygesen et al. 2010; Yang et al. 2018). Tai et al. (2020) reported the appearance of middle lamellae (ML) as linear structure in the case of spruce (historical) wood at 830 nm and 1064 nm excitation wavelengths, respectively and fluorescence intensity was stronger owing to the high (70%) lignin content.

Figure 4 illustrates the effect of laser power (at 710 nm EX WL) on the enhancement of the emission (fluorescence) signal of lignin. Keep in mind that wood cell wall is comprised of lignin, hemicellulose, and cellulose, all of which can exhibit autofluorescence in solid-state. Based on our earlier fluorescence “*fingerprint*” research of lignocellulosic fiber, (Hoque et al. 2023) at > 290 nm EX WL (UVB light), fluorescence crosstalk from polysaccharides is possible to minimize. In other words, longer EX WL is suitable for lignin, although upon 800, and 900 nm EX WL, emission from lignin became weaker (Figure S4). This observation is somewhat different from Tai et al. (2020) which could be due to the lower lignin content (~ 30.0 wt%) in cedar (Takada and Saka 2015).

In the case of natural wood (top micrographs), upon increasing the laser power (0.5–2.0%), the brightening of lamella structure became apparent, and at low laser power (0.5%), lignin emission was very weak, visualized as underexposed (dark) micrograph. On the other hand, in DES-treated wood (bottom micrographs), even at low laser power (0.5%), strong lignin emission was observed, and with its increment, the detector was saturated with emission signal, visualized as over-exposed (bright) micrographs. Such a stark contrast could stem from DES-aided lignin diffusion, enhancing the emission signal.

Thus, by leveraging the high light collection efficiency of 2PEM (in NDS-detection), coverage of lignin on the subsurface of DES-treated wood was confirmed, supporting the morphological analysis by SEM (Fig. 3). However, under 710 nm EX WL, cellulose could still contribute to the emission; hence, we performed multi-channel two-photon imaging (less efficient than NDS-mode), which are 435–485 nm (ch1: Blue), 480–520 nm (ch2: cyan), and 500–550 nm (ch3: green), respectively.

Figure 5 depicts the variation in emission intensity of wood samples under these three different channels, which can be visualized as the micrographs (left side) and the corresponding bar chart (right side) provide a semi-quantitative perspective. Notably, “green” channel emission (average intensity) was lowest for NW (untreated) specimen while it was highest for DES-treated wood — lignin emission is stronger in ch3 (wavelength > 500 nm) as reported by Kapsokalyvas et al. (2020) for corn stover, Hoque et al. (2023) for unbleached kraft pulp, to name a few.

Overall, both LSCM (high-efficiency mode, but qualitative) and 2PEM (low-efficiency mode, but semi-quantitative) characterization revealed spatial redistribution of lignin from the inner wood structure to the subsurface of natural wood upon DES treatment, which provides the chemical information by leveraging lignin autofluorescence. Such localization of lignin on wood surfaces can render interesting physico-chemical features (like water resistance) upon densification.

### Mechanical characterization: modulus of rupture (MOR) evaluation

**Fig. 6:**
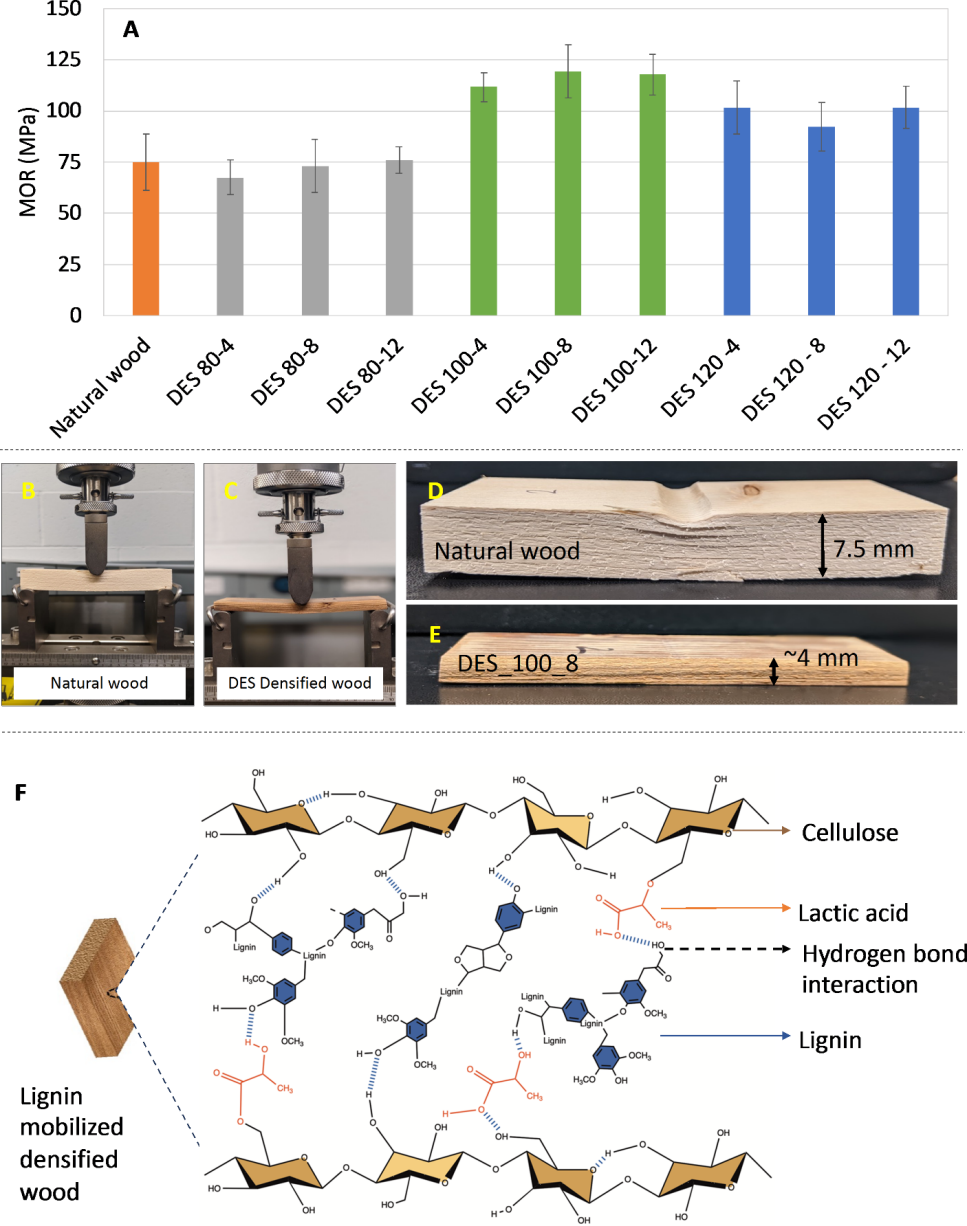
Mechanical (3-point bending) characterization of natural and DES-treated wood. **A**- (bar graph): Variation in the modulus of rupture (MOR) at various temperatures and time combinations. **B, C** (digital photographs): 3-point bending tests done by the universal testing machine on natural and DES-densified wood, respectively. **D, E** (digital photograph): Images showcasing qualitative hardness of natural and DES-densified wood after yielding, respectively. **F** (scheme): Proposed mechanism of lignin participation in forming additional hydrogen bond interactions (possibly) between adjacent cell wall’s hydroxyl group. (Supplementary info: Figure S1 – FTIR spectrum shows a characteristic peaks at 1598, 1510, and 1452 cm^-1^, corresponding to the vibration of the aromatic skeleton structure in lignin and hydroxyl stretching vibration at 3350 cm^-1^; 1729 cm^-1^ corresponding to the carbonyl (C = O) stretching

The MOR shows the maximum stress a material could bear before it yields. It was calculated using an Instron universal testing machine in compression mode with a head speed of 10 mm/min (Fig. 6B, C). As mentioned in the procedure, the bending strength of DES-treated densified wood for a combination of three different temperatures for DES treatment and three different time spans for DES treatment was analyzed. Figure 6A shows the MOR values of samples treated with three different treatment temperatures (80, 100, 120) and at three different periods (4, 8, and 12 h).

Upon conducting a statistical analysis, we found there are no significant differences in MOR results among the DES-80 samples when compared to natural wood. This implies that these specific treatments did not lead to a noticeable change in MOR properties compared to untreated natural wood samples. This outcome is likely because the low temperature of the DES treatment was insufficient to solubilize lignin, as corroborated by Ran et al. (2023). In their study, it was found that the dimensional stability of DES-treated specimens at 90 °C for 14 h was exceptional. Our results align with this finding, suggesting that the low treatment temperature of 80 °C was not adequate to induce significant changes in the wood’s structure. Additionally, the high viscosity of DES compounds, due to their extensive hydrogen-bond network structure, hinders mass transfer efficiency (Mišan et al. 2020). This limitation further reduces the effectiveness of lignin solubilization and subsequent crosslinking with cellulose during the densification process. Consequently, the minimal crosslinking network formed at 80 °C did not substantially alter the MOR properties.

As the temperature of DES increased from 80 °C to 120 °C, the rate at which lignin solubilizes also increased. This is due to the ionization of lactic acid providing more hydrogen ions for choline chloride, accelerating the breaking of ether bonds and solubilizing more lignin (Mišan et al. 2020). However, excessively high treatment temperatures (e.g., 120 °C) can lead to the over-removal of lignin, which acts as a binder in wood. This over-removal disrupts the interconnection between wood fibers, resulting in low cell bonding strength, a loose structure, and decreased mechanical properties compared to DES-100 samples (Song et al. 2018b).

On the contrary, too little lignin dissolution results in a low amount of exposed cellulose hydroxyl groups. The lack of sufficient lignin dissolution can prevent effective bonding and integration of cellulose fibers, leading to poor structural integrity and weaker mechanical properties. Therefore, finding an optimal treatment temperature is crucial to balancing lignin solubilization and retention, to maintain or enhance the mechanical properties of treated wood. It was observed that the DES-treated wood samples at 100 °C had better MOR values (~ 120 MPa) compared to wood samples treated at 80 °C and 120 °C as well as natural wood (75 MPa), which was almost 60% higher. One of the main reasons for the rise in MOR is likely attributed to the in-situ formation of the crosslinking network that occurs between the regenerated lignin and cellulose during the densification process (Ran et al. 2023). A previous study on the densification of water-saturated wood by Felhofer et al. (2020) also reported lignin dislocation at ambient temperature under pressure. However, in our study, we are using temperature-assisted densification, which makes it easier for lignin to mobilize. Visually, lignin mobilization (from inner structure to surface) was observed using optical microscopy (Figs. 4 and 5). Song et al. (2018b) developed the densified wood with a specific strength higher than metal alloys, which was speculated to be due to the increase in hydrogen bonding interaction between cellulose fibers after densification. However, in our study, we speculate that the increase in MOR of the densified wood is not just due to the hydrogen bond interaction between the hydroxyl groups of cellulose but also due to the DES treatment, which led to better lignin-induced adhesion between cellulose-lignin, as well as cellulose-lactic acid-lignin polymers (Fig. 6F). A similar effect has been reported in the previous literature as well (Guo et al. 2022; Ran et al. 2023; Xia et al. 2021). This is in line with the FTIR spectrum (Supplementary info, Figure S1), which indicates an increase in the absorption peak intensity corresponding to the lignin skeleton structure in the DES-treated wood compared to the natural wood. To see the effect of DES on the crystallinity of wood, we performed XRD, and Figure S5 shows the XRD pattern for the natural wood and this work (DES-impregnated and oven-treated) sample. It can be observed from Figure S5 that X-ray diffraction (XRD) patterns of the natural wood powder, and DES – oven (this work) exhibited similar diffraction peaks (2*θ* = 16.2° (101), and 22.5° (002), and 34.7° (040) (Cheng et al. 2011) corresponding to lattice plane of cellulose I polymorph. These similarities in the peaks implies that the DES treatment did not significantly change the crystal structure of cellulose I compared to natural wood. However, there was a significant increase in the Cr*I* for the DES-treated wood compared to natural wood (35.66%) because while preparing the samples for XRD, both samples were washed using ethanol to remove DES chemical after the treatment, which also led to the removal of some amorphous components of wood (for e.g. hemicellulose and lignin) and also likely due to the removal of the amorphous regions of cellulose (Almeida et al. 2022).

A statistical model (two-way ANOVA) was fit using R studio (Team 2020) to understand the effect of individual variable time, and temperature as well as the combined effect of time and temperature. It was evident from the ANOVA Table 1 that temperature had a significant effect (*p* < 0.05) on the MOR values (response variable), whereas the combined effect of time and temp (independent variable), as well as the individual effect of time, had not significant impact on the MOR values. The positive characteristics, i.e., increase in MOR showcased here, indicate that wood densified with DES assistance, produced using recyclable DES and sustainable wood, has the potential to replace traditional synthetic engineering materials like concrete and steel structures.

While calculating MOR, the qualitative hardness of the original and DES-treated densified wood (DES-100-8) was also assessed. As expected, it was apparent from Fig. 6D, E that after the samples yielded in the three-point bending test, natural wood had a bigger and wider indentation on the surface compared to the DES-densified wood. So, densification combined with DES improved the dimensional stability and surface hardness of the composite compared to natural wood.

**Table 1:** Two-way ANOVA test to understand the effect of individual variable time, and temperature, as well as the combined effect of time and temperature on the MOR of the DES, treated wood with three different temperatures (80, 100, 120 °C) and three different time intervals (4, 8, 12 h). DF: degree of freedom; Sum sq: measure of the variability within each factor and the residuals; Mean Sq: average variation or variance among the sample means; F value (F-statistic): it tests the null hypothesis that the means of the groups are equal. Higher F values suggest that the factor may be significant; pr (> F): this column represents the p-value associated with the F-statistic. Lower p-values indicate stronger evidence against the null hypothesis

	DF	Sum Sq.	Mean Sq.	F Value	Pr (> F)
Time	2	18.5	9.2	0.0101	0.98996
Temp	2	8630.7	4315.4	4.7192	0.02095
Time: Temp	4	415.3	103.8	0.1135	0.97627
Residuals	20	18288.5	914.4		

### Mechanical characterization: compression shore D hardness and springback

**Fig. 7:**
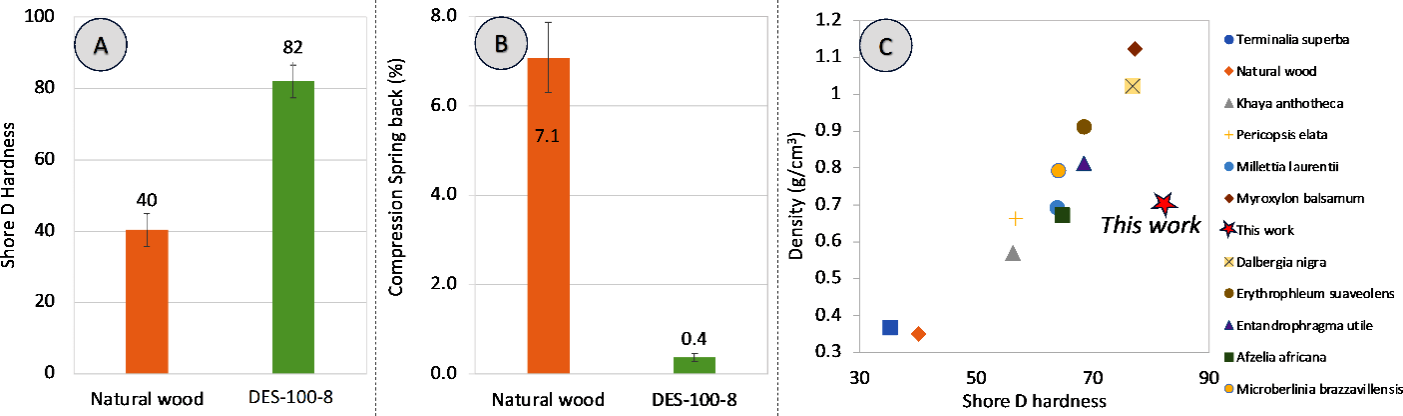
Mechanical (Shore D hardness and springback) characterization of natural and DES-treated wood. **A** (bar graph): Variation in Shore D hardness; **B** (bar graph) compression springback (CS) for natural and DES-densified wood (DES-100-80) immediately after 8 h of compression; **C** (correlation plot): Shore **D** hardness of DES-100-8 (this work) compared to commercially available hardwoods (Esteves et al. 2021)

Hardness was assessed qualitatively while performing the MOR test. However, to quantitatively compare the hardness of natural wood and DES-treated wood, the Shore D hardness test was carried out. It was observed (Fig. 7A) that the surface hardness improved significantly after the DES treatment, followed by densification. Shore D hardness of DES-100-8 improved by a factor of 2 compared to the natural wood (from 40 to 82). Moreover, the DES-densified wood exhibited the highest hardness compared to other hardwoods with similar or higher density (Fig. 7C). For instance, hardwoods such as Sipo (*Entandrophragma utile*), Tali (*Erythrophleum suaveolens*), and Zebrano (*Microberlinia brazzavillensis*) which have a density higher than the DES-densified wood in the range of (0.8–1.0 g/cm^3^) showcased shore hardness of 68.4, 68.5, and 64.20, respectively (Esteves et al. 2021). The wood with higher densities (> 1.0 g/cm3), such as Santos (*Myroxylon balsamum*) and Rose (*Dalbergia nigra*), also exhibited lower shore hardness values of 77.20 and 76.80, respectively (Fig. 7C) (Esteves et al. 2021).

The compression springback (CS) was calculated as the ratio of the difference of actual thickness taken immediately after compression and target thickness to the differnce of uncompressed wood sample and target thickness (spacer 4 mm), expressed as a percentage. It was evident from the results obtained (Fig. 7B) that the DES-100-8 (DES-densified wood) had a compression springback of just 0.4%, whereas the directly densified natural wood had 7.1%, indicating that the DES treatment accelerated the relaxation of internal stress (Kariz et al. 2017). This may be because of the breakage of molecular chains, especially in lignin, which got reformed during the temperature-assisted densification step (Fig. 6F).

In contrast to the traditional delignification technique, DES-densified wood (in this study) is produced by consuming less chemicals and producing less waste (more sustainable). Moreover, the current approach affords the retention of the maximum lignin content in the wood, which further acts as a binder during densification. Thus, less lignin waste is generated in this greener process as safer chemicals such as choline chloride and lactic acid are used, and after the washing step to remove excess DES, this DES can be recycled back (Amesho et al. 2023; Ran et al. 2023; Xia et al. 2021; Xu et al. 2020). Supplementary data Figure S2 shows the amount of lignin remaining after the DES treatment using 2 methods: (1) Boiling the wood block in DES solution and (2) Impregnating DES solution in the block and oven heating. Lignin content in the case of boiling dropped by 20% compared to the natural wood (from 28.2 to 22.7%), which was contradictory to the previous literature, which claimed to retain most of the lignin (Ran et al. 2023). In this work, we used oven heating, which reduced the leaching of lignin significantly, around 2.5% (from 28.2 to 27.5%). Hence, an additional DES recycling step was not needed, saving energy costs and processing time.

### Surface hydrophobicity characterization: Contact angle focused

**Fig. 8:**
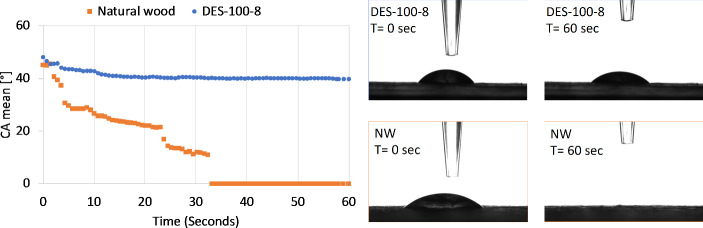
Surface hydrophobicity characterization (sessile drop test) of natural wood (NW) and DES-treated wood (DES-100-8). Digital images on the right show the droplet images at *t* = 0 s and *t* = 60 s for the DES-100-8 and NW

**Fig. 9:**
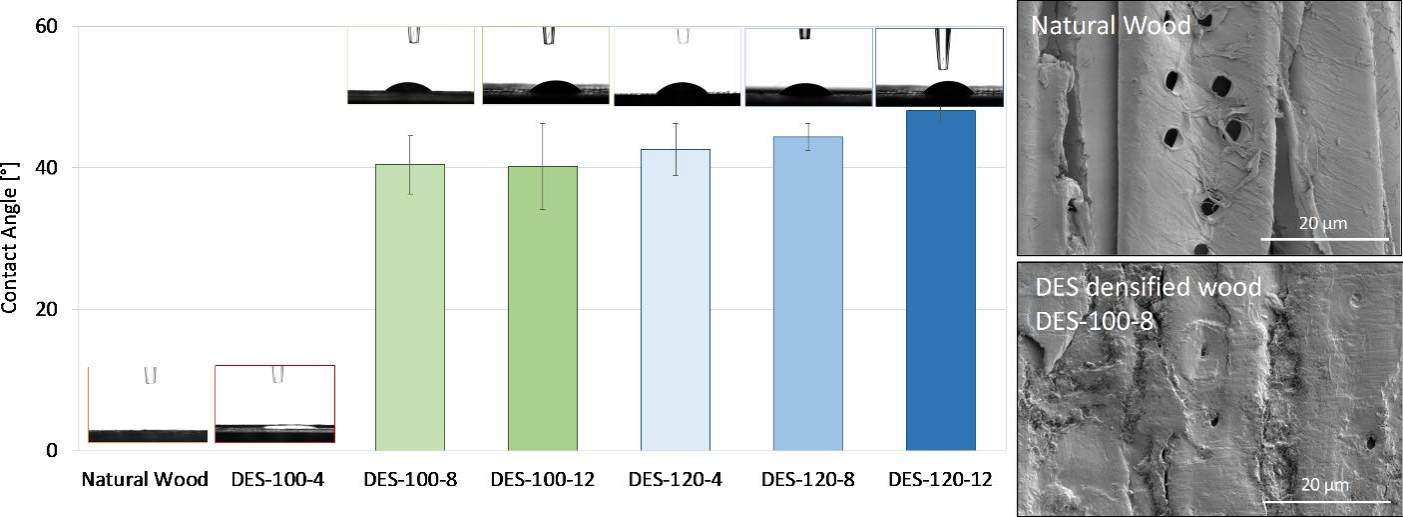
Surface hydrophobicity (sessile drop test) characterization of natural and DES-treated wood as a function of DES treatment (100–120 °C) and duration of (4–12 h). (Left side) Bar chart of contact angle values and (right side) SEM micrographs of the surface before (natural wood) and after the DES treatment and densification (DES-100-8)

A sessile drop test was performed on the surface to study the water-repellency property of the DES-treated densified wood. It was observed that the contact angle was significantly higher in the DES-treated wood compared to the original wood (Fig. 8). The contact angle for the original sample (natural wood) decreased from 43° to 0° in just 30 s. On the contrary, for the DES-100-8 sample, it decreased slightly but stabilized around 41°. This test was performed on other samples, including densified samples, which were DES-treated at different temperatures and at different times. Figure 9 showcases the mean contact angle for DES-100-4, DES-100-8, DES-100-12, DES-120-4, DES-120-8, and DES-120-12. The DES-treated densified sample at 100 °C and 4 h (DES-100-4) did not have any contact angle as the water droplet was absorbed by the wood, as shown in Fig. 9 and also shown in Figure S3B. A similar trend was observed for the DES-80 samples (Figure S3).

**Fig. 10:**
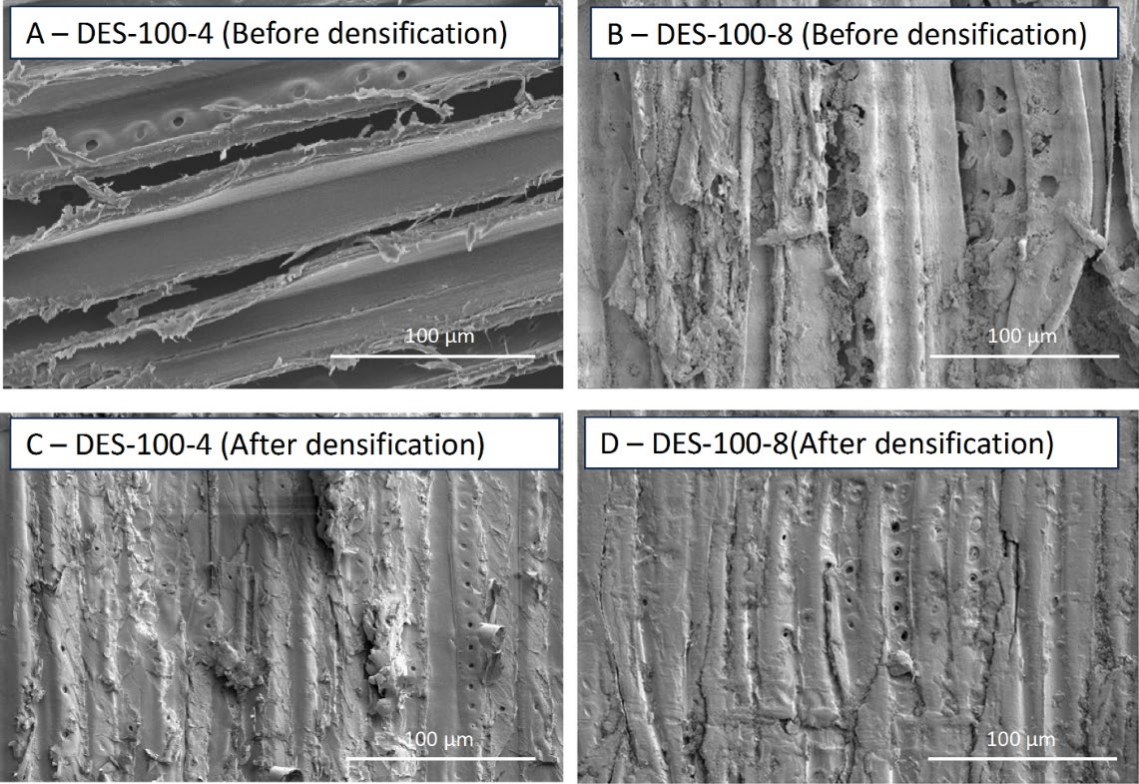
Surface SEM micrographs at low (500x) magnifications for (**A**) DES-100-4 (Before densification); (**B**) DES-100-8 (Before densification); (**C**) DES-100-4 (After densification); (**D**) DES-100-8 (After densification)

One of the possible reasons for the decrease in water absorption for DES-100-8 is due to the collapse of pores (pits) on the surface of wood, as shown in SEM images (Fig. 9 Right). A similar observation was also reported in our previous work (Gondaliya et al. 2023a, b). Furthermore, SEM surface images (Fig. 3) reveal that lignin adheres to the cell walls following DES treatment and regeneration. This is further corroborated by Fig. 10, which shows that, unlike DES-100-8, DES-100-4 does not exhibit regenerated lignin precipitation on the surface prior to densification post-DES treatment. This suggests that the enhanced water resistance of DES-100-8, compared to natural wood, is also due to the formation of crosslinking networks between wood components, such as regenerated lignin and cellulose (Sandberg et al. 2021). The regenerated lignin exhibits amphiphilic properties owing to the presence of both a non-polar hydrophobic backbone (including hydrocarbon groups and phenylpropane), and polar hydrophilic side chains (such as phenolic hydroxyl groups) (Xia et al. 2021). This amphiphilic nature is advantageous for concurrently attaining robust mechanical strength and water stability. The polar hydrophilic side chains can form crosslinks with cellulose micro/nanofibrils (Fig. 6F), enhancing mechanical strength, while the non-polar hydrophobic backbone serves to impede water permeation (Xia et al. 2021).

## Conclusion

A sustainable and economical process was developed to synthesize densified wood with enhanced mechanical performance (MOR and surface hardness) as well as water-repellency properties. Herein, we demonstrated a synergistic combination of simultaneous temperature-assisted physical modification with in-situ chemical modification using regenerated lignin. Statistically, we saw temperature had a significant effect on the mechanical performance of DES-treated densified wood, and 100 °C was an ideal temperature for the treatment. The DES-treated wood showed excellent mechanical properties (MOR: >50% improvement), surface hardness (improved by almost 100%), better shape fixation (low set recovery), and water-repellency properties compared to natural wood. By utilizing the inherent lignin inside the wood, less lignin waste was generated, and after the DES treatment, the regenerated lignin acted as a glue to aid the densification process, which led to a reduction in densification time and energy compared to the traditional approach. This strategy has the potential to be commercially used at an industry scale for advanced engineering applications, such as house cladding, construction, aerospace, and even shipment containers. Future work will be focused on understanding the effect of different DES combination systems on the properties of densified wood.

## Electronic supplementary material

Below is the link to the electronic supplementary material.

Supplementary Material 1

## Data Availability

No datasets were generated or analysed during the current study.
