# Supplementary material for: Green and sustainable fabrication of DES-pretreated high-strength densified wood
Source: Wood Sci Technol. 2024 Aug 27;58(5-6):1901–23. doi: 10.1007/s00226-024-01594-7 (PMC11493799; doi:10.1007/s00226-024-01594-7)
Supplement: Supplementary file 1 — Supplementary Material 1 [file 226_2024_1594_MOESM1_ESM.docx]

**Green and sustainable fabrication of DES-pretreated high-strength densified wood**

Akash Gondaliya^a^, Mahfuzul Hoque^a^, Sreenath Raghunath^a^, E. Johan Foster*^a^

*^a^Chemical and Biological Engineering, University of British Columbia, Vancouver, Canada. E-mail:*[*johan.foster@ubc.ca*](mailto:johan.foster@ubc.ca)

**Supplementary information**

## **Attenuated Total Reflectance-Fourier Transform Infrared Spectroscopy (ATR-FTIR) Analysis**

Figure S1: ATR-FTIR spectra of natural wood (cedar) depicted in a solid blue line and DES-densified wood denoted by the solid orange line. The characteristic peak is highlighted by the black dotted line at 1598, 1510, and 1452 cm^-1^, which corresponds to the vibration of the aromatic skeleton structure in lignin and hydroxyl stretching vibration at 3350 cm^-1^; 1729 cm^-1^ corresponding to the carbonyl (C=O) stretching.

ATR-FTIR was conducted for the natural wood powder as well as DES-treated densified wood to investigate further the chemical group changes occurring during the lignin regeneration process. The FTIR spectrum (Figure S1) of DES-treated wood composite attributed the absorption band at 1598, 1510, and 1452 cm^-1^ which corresponds to the vibration of the aromatic skeleton structure in lignin and hydroxyl stretching vibration at 3350 cm^-1^(Li et al., 2016; Tian et al., 2020). Additionally, these peaks were not prominent in the control wood samples, suggesting that the lignin regeneration and migration were happening in the DES-treated wood. This was also evident from the optical microscopy images (confocal and two-photon) and SEM results. Moreover, a sharp peak was observed at 1729 cm^-1^ corresponding to the carbonyl (C=O) stretching indicating the partial esterification of the hydroxyl group of cellulose by lactic acid during the densification treatment of DES-treated wood samples (Wang et al., 2020).

## **Lignin Content**

To determine the lignin content after the DES treatment, Klason test was performed for the natural wood (original lignin content), DES-oven (this work), and DES boil wood. It was evident from Figure S*2*, that the boiling leaches away lignin and there was a reduction of lignin content of almost 19% compared to the natural wood samples. Whereas in our approach we intend to keep most of the lignin inside the wood to reduce the lignin waste. There was a reduction of 2.5% in the DES oven sample because of the washing step before densification.

Figure S2: Lignin content % calculated for the natural wood (NW); DES-treated wood via boiling the wood in DES solution (DES-Boil); DES-treated wood via vacuum impregnation and oven heating (DES-Oven)

## **Contact Angle Test**

Figure S3: Contact angle measurements for DES-treated densified wood at 80 °C and 100 °C. The contact angle improved after DES treatment was done at 100 °C and for 8 hours.

## **Effect of Excitation Wavelength on Wood Surface**

The wavelength of 710 nm was selected based on the assessment done to observe the ideal excitation wavelength for the lignin fluorescence in the wood as shown in Figure S4

Figure S4: Natural wood excited with three different wavelengths, which are 710, 800, and 900 nm, to observe the fluorescence of lignin in wood.

## **Crystallinity Index**


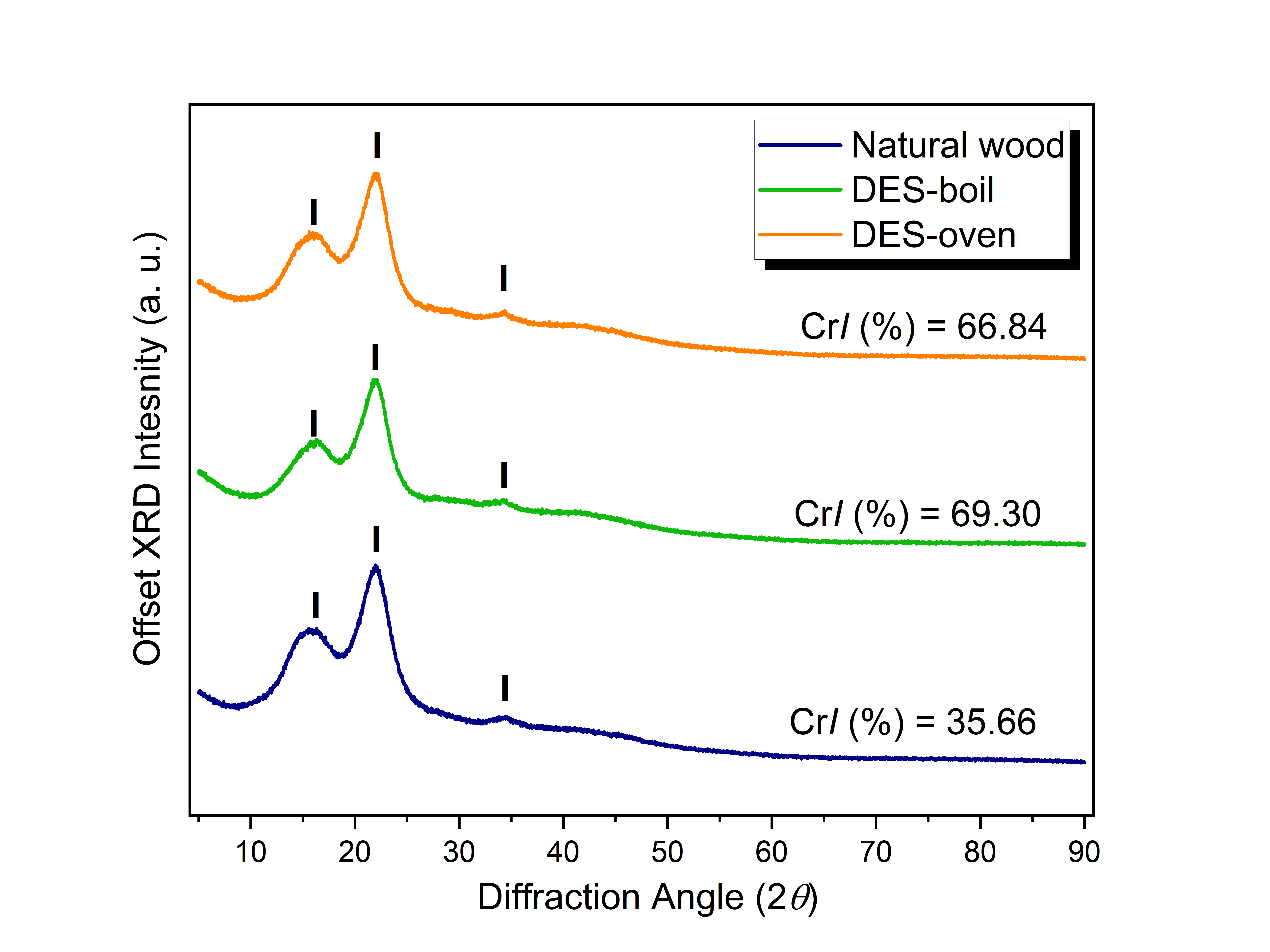


Figure S5: Powder XRD diffractograms for the Natural wood, DES-boil (wood soaked and boiled in DES solution), and this work, labeled DES-oven (wood impregnated with DES and oven heated without boiling). Cellulose Iα powder diffraction file (PDF) no. is 00–056–0179. Crystallinity index (CrI) or crystallinity (in %) was calculated using the peak-height method (Thygesen et al., 2005).

Table S1: Crystallinity index of Natural and DES treated wood calculated via Segal method.

| **Sample** | **Preparation Method** | **Crystallinity Index (CrI) %** |
| --- | --- | --- |
| Natural Wood | Natural wood was powdered finely | 35.66 |
| DES – Boil | Natural was boiled in DES solution for 8 hours at 100 °C and then washed with ethanol to remove excess DES and then powdered | 69.30 |
| DES – Oven | Natural was impregnated with DES solution and treated via oven heating for 8 hours at 100 °C and then washed with ethanol to remove excess DES and then powdered | 66.84 |

**Physical data:**

Table S2: Data comparing the physical characteristics of Natural and densified wood. All values are the mean of 5 replicates.

| Sample | L | B | H | Density |
| --- | --- | --- | --- | --- |
| Cedar Species | mm | mm | mm | g/cc |
| Natural wood | 100 | 38 | 7.5 | 0.45 |
| Densified wood DES -100-8 | 100 | 40 | 4 | 0.74 |

**References:**

Li, Y., Liu, Y., Chen, W., Wang, Q., Liu, Y., Li, J., & Yu, H. (2016). Facile extraction of cellulose nanocrystals from wood using ethanol and peroxide solvothermal pretreatment followed by ultrasonic nanofibrillation. *Green Chemistry*, *18*(4), 1010–1018. https://doi.org/10.1039/C5GC02576A

Thygesen, A., Oddershede, J., Lilholt, H., Thomsen, A. B., & Ståhl, K. (2005). On the determination of crystallinity and cellulose content in plant fibres. *Cellulose*, *12*(6), 563–576. https://doi.org/10.1007/S10570-005-9001-8/METRICS

Tian, D., Guo, Y., Hu, J., Yang, G., Zhang, J., Luo, L., Xiao, Y., Deng, S., Deng, O., Zhou, W., & Shen, F. (2020). Acidic deep eutectic solvents pretreatment for selective lignocellulosic biomass fractionation with enhanced cellulose reactivity. *International Journal of Biological Macromolecules*, *142*, 288–297. https://doi.org/10.1016/j.ijbiomac.2019.09.100

Wang, H., Li, J., Zeng, X., Tang, X., Sun, Y., Lei, T., & Lin, L. (2020). Extraction of cellulose nanocrystals using a recyclable deep eutectic solvent. *Cellulose*, *27*(3), 1301–1314. https://doi.org/10.1007/S10570-019-02867-2
